# Improvement of Gait Biomechanics after Endovascular Therapy for Patients with Intermittent Claudication Associated with Aortoiliac Occlusive Disease

**DOI:** 10.3400/avd.oa.25-00006

**Published:** 2025-06-10

**Authors:** Norinobu Ogasawara, Takaaki Kakihana, Daijirou Akamatsu, Yuta Tajima, Michihisa Umetsu, Takanori Ishida, Michiaki Unno, Hitoshi Goto, Takashi Kamei, Masahiro Kohzuki

**Affiliations:** 1 Department of Surgery, Tohoku University Graduate School of Medicine, Sendai, Miyagi, Japan; 2 Biodesign Department, Translational Research Center, The University of Tokyo Hospital, Tokyo, Japan; 3 Department of Internal Medicine and Rehabilitation Science, Tohoku University Graduate School of Medicine, Sendai, Miyagi, Japan; 4 Yamagata Prefectural University of Health Sciences, Yamagata, Yamagata, Japan

**Keywords:** EVT, gait analysis, LEAD, PAD

## Abstract

**Objectives:** Gait disturbances increase mortality rates in lower extremity artery disease. Changes in gait biomechanics after endovascular therapy for intermittent claudication associated with lower extremity artery disease remain unknown. This prospective study investigated the effect of endovascular therapy on gait biomechanics in intermittent claudication.

**Methods:** We recruited 10 patients (14 affected limbs) with intermittent claudication caused by isolated aortoiliac artery lesions who underwent endovascular therapy, and 10 healthy controls. Using 3-dimensional motion analysis, we measured biomechanical gait parameters preoperatively and over 6 months postoperatively, comparing them with those of healthy controls.

**Results:** One month after endovascular therapy, parameters improved significantly compared with preoperative values: step length (preoperative median 52.47 [interquartile range 47.11, 60.33]–postoperative 58.53 [54.63, 64.54] cm; *P* < 0.0037), walking speed (90.17 [73.98, 108.9]–103.49 [97.66, 117.94] cm/s; *P* = 0.0022), hip flexor moment (−0.75 [−1.04, −0.51] to −0.94 [−1.07, −0.74] Nm/kg; *P* = 0.04), and pull-off power generated by hip flexor muscles (H3, 0.68 [0.38, 1]–0.86 [0.72, 1.1] W/kg; *P* = 0.018). Preoperative joint power declined significantly compared to control parameters. However, 6 months postoperatively, no significant differences were observed.

**Conclusions:** Endovascular therapy for isolated aortoiliac artery lesions improved biomechanical gait parameters in patients with intermittent claudication.

## Introduction

Intermittent claudication (IC) is the most common symptom of lower extremity artery disease (LEAD). IC limits physical function, causes poor quality of daily life (QOL), reduces activities of daily living,^1)^ and is associated with high mortality in patients with LEAD.^2–4)^

Three-dimensional (3D) motion analysis can calculate biomechanical parameters such as joint angles, angular velocity, joint moments, and joint power, and can estimate muscle function related to gait.

3D motion analysis is often used in the field of medical rehabilitation, but it is also useful for accurately understanding IC as a gait disturbance.^5–7)^ Recently, there have been reports evaluating the therapeutic effects of medical treatment,^8)^ and supervised exercise treatment^9,10)^ for patients with IC using 3D motion analysis. In our previous study, we also reported that patients with IC associated with isolated aortoiliac artery lesions showed dysfunction of the hip flexor (HF) muscle in particular, as assessed by 3D motion analysis.^11)^

We usually observed that IC improves after endovascular therapy (EVT); however, the detailed changes in gait status are not well known. Therefore, we conducted a prospective cohort study using 3D motion analysis to examine the effect of EVT on biomechanical gait parameters in patients with aortoiliac artery lesions.

## Patients and Methods

### Participants

We recruited patients with IC associated with isolated aortoiliac artery lesions who visited Tohoku University Hospital between January 2016 and June 2020. Patients were considered eligible for EVT if they had an ankle brachial index (ABI) <0.9 and significant stenosis or occlusion of the aortoiliac artery. Significant stenosis was defined as a peak systolic velocity ratio >2 on duplex ultrasound and/or >50% stenosis by computed tomography angiography. Patients with chronic limb-threatening ischemia and those with limited walking ability for reasons other than IC, such as pulmonary, cardiac, nervous, and musculoskeletal diseases, were excluded.

Healthy controls were recruited from the community to compare biomechanical gait parameters. Controls were defined as people with no symptoms and an ABI >1.0, and were matched with patients according to gender, age, height, and mass.

All participants gave written informed consent before being included in the study.

The study protocol was approved by the Tohoku University Ethics Committee (approval number: 2014-1-718).

### EVT

EVT was performed by puncturing the unilateral or bilateral common femoral artery under local anesthesia. The degree of stenosis, lesion length, vessel size, and characteristics were evaluated by angiography and intravascular ultrasonography (IVUS). Pre-dilatation with a balloon was performed if required. In all cases, a bare metal stent or stent graft was positioned to cover the entire length of the lesion, followed by sufficient post-dilatation with a balloon. Device fitting and residual stenosis were confirmed by angiography and IVUS. Technical success was defined as residual stenosis <30%. Intraoperative complications were defined as major bleeding, flow-limiting dissection, and thromboembolism, while postoperative complications were defined as major bleeding, pseudoaneurysm, and wound infection.

### Follow-up

Measurements were taken before surgery and compared with follow-up values at 1 week, 1 month, and 6 months after EVT, as described below. During the follow-up period, we encouraged exercise but did not provide instructions for any structured exercise, and patients did not undergo pre- or postoperative rehabilitation. Patients’ physical activity levels were not monitored.

### Measurements

#### Gait biomechanics

We measured participants’ biomechanical gait parameters in the rehabilitation center of Tohoku University Hospital. Spatiotemporal and kinetic parameters were measured with an 8-camera MAC 3D motion analysis system (Motion Analysis, Santa Rosa, CA, USA) at a sampling rate of 120 Hz. Reflective markers were attached to 17 anatomical landmarks on the participants, as suggested by The Clinical Gait Analysis Forum of Japan:^12)^ the right and left acromion, anterior and posterior superior iliac spine, greater trochanter, femoral lateral epicondyle, lateral malleolus, 5th metatarsal head, calcaneus, and the right angulus inferior scapulae (**Supplemental Fig. 1**).

Four 90 × 60 cm force plates (Anima, Tokyo, Japan) were used to measure the ground reaction force at a sampling rate of 1200 Hz.

Participants walked barefoot along a 7-m walkway at a self-selected speed. If patients felt claudication pain, they rested until the pain improved, and these data were excluded from the study (in fact, few data were excluded). Mean values of data from 5 trials were used for analysis.

#### Biomechanical data analysis

Biomechanical data were filtered using a low-pass filter with a 20 Hz cutoff frequency. The spatiotemporal parameters, such as step length, cadence, and walking speed, and kinetic parameters, including joint moment and joint power of the lower limb in the sagittal plane during the stance phase, were calculated using KineAnalyzer version 4.0.3.1409 (Kissei Comtec, Nagano, Japan). The peak joint moment was calculated for the hip extensor (HE), HF, knee extensor (KE), knee flexor (KF), ankle dorsiflexor (AD), and ankle plantar flexor (AP). Joint power was calculated by joint moment × angular velocity. The peak joint power was calculated along the phase defined by Eng and Winter.^13)^ Hip power generation during loading response by concentric contraction of the HE muscle (H1), hip power absorption during mid stance by eccentric contraction of the HF muscle (H2), hip power generation during preswing by concentric contraction of the HF muscle (H3), knee power absorption during loading response by eccentric contraction of the KE muscle (K1), knee power generation during mid stance by concentric contraction of the KE muscle (K2), knee power absorption during preswing by eccentric contraction of the KE muscle (K3), ankle power absorption during mid stance and terminal stance by eccentric contraction of the AP muscle (A1), and ankle power generation during preswing by concentric contraction of the AP muscle (A2) (**Supplemental Fig. 2**).

#### Treadmill test and recovery time

Before walking, near-infrared spectroscopy probes (NIRO-200NX; Hamamatsu Photonics, Shizuoka, Japan) were attached to the bilateral medial head of the gastrocnemius muscle,^14)^ and baseline tissue oxygenation data were recorded. A treadmill test was then performed at a grade of 12% and a speed of 0.5 or 0.67 m/s. The choice of speed was determined by each patient’s walking ability and remained consistent during the follow-up period. Pain-free walking distance (PWD) and maximum walking distance (MWD) were recorded during a maximum of 10 min of treadmill walking. After the treadmill test, the patients sat on a chair to rest for 5 min, and the recovery time (RT) was recorded; RT was defined as the time for tissue oxygenation levels to return to baseline.^15,16)^

#### ABI

ABI was measured at each follow-up scheduled time point in the clinical physiological laboratory of Tohoku University Hospital.

#### Walking Impairment Questionnaire (WIQ) score

QOL was evaluated using the WIQ^17)^ at the preoperative, 1-month, and 6-month visits. The WIQ consists of four items: walking pain, walking distance, walking speed, and stair climbing. Each item was scored on a scale of 0–100 points, with a lower score indicating more restricted walking. The WIQ includes questions about walking during the past week and was not applied at the 1-week visit, as the patients may not have walked sufficiently for several days after surgery.

### Statistics

Categorical variables were expressed as numbers and percentages. Many continuous variables did not follow a normal distribution according to the Shapiro–Wilk test, so all continuous variables are expressed as median (interquartile range [IQR]), and we used nonparametric statistical methods. Fisher’s exact test was used for categorical variables, and the Mann–Whitney *U*-test was used for continuous variables.

Time-series data for ABI, WIQ, PWD, MWD, RT, and biomechanical parameters were analyzed using pairwise Friedman rank-sum tests. If the Friedman test showed a significant difference, the Wilcoxon signed-rank tests were used for multiple pairwise comparisons between each pair of measurements (preoperative/1 week, preoperative/1 month, preoperative/6 months, 1 week/1 month, 1 week/6 months, and 1 month/6 months) with the Bonferroni *P* value adjustment method.

All statistical analyses were performed with EZR version 1.54 (Saitama Medical Center, Jichi Medical University, Saitama, Japan).^18)^ Statistical significance was defined as *P* < 0.05.

## Results

### Participants

Fourteen patients with IC associated with isolated aortoiliac artery lesions were recruited for this study, but 4 dropped out (2 withdrew consent, 1 developed acute lumbago, and 1 could not visit due to the COVID-19 pandemic) at the 1- or 6-month time points. Ultimately, 10 patients (14 limbs, including 6 patients with unilateral lesions and 4 patients with bilateral lesions) completed all measurements and were included in the study. There were no differences in characteristics, including gender, age, height, mass, ABI, and comorbidities, between patients who completed all measurements and those who did not (data not shown).

All baseline parameters (walking distance, WIQ, and gait biomechanics) showed no significant differences between patients with unilateral and bilateral lesions (data not shown). Therefore, we analyzed 14 limbs, including both patients with unilateral and bilateral lesions, in this study.

We recruited 10 matched healthy controls, as mentioned above, to compare biomechanical gait parameters.

### Characteristics, EVT, and follow-up

Characteristics of the patients and controls are shown in **Table 1**. There were no significant differences in comorbidities between the groups.

**Table:** Table 1 Characteristics of patients and controls

	Patients (n = 10)	Controls (n = 10)	*P* value
Male gender	9 (90)	8 (80)	1
Age (years)	72 (68, 73)	71 (71, 74)	0.88
Height (m)	1.62 (1.6, 1.7)	1.65 (1.61, 1.66)	0.85
Mass (kg)	60.1 (54.6, 62.9)	59.2 (54.1, 64.7)	0.94
Hypertension	8 (80)	6 (60)	0.63
Dyslipidemia	6 (60)	5 (50)	1
Diabetes mellitus	2 (20)	0 (0)	0.47
Coronary artery disease	1 (10)	0 (0)	1
Cerebrovascular disease	2 (20)	0 (0)	0.47
Current or past history of smoking	9 (90)	5 (50)	0.14

Data are presented as n (%) or median (interquartile range).

Characteristics of the symptoms of each affected limb and arteriosclerosis lesions are summarized in **Table 2**. Of the affected limbs, 79% had a Fontaine classification of IIa and 21% were classified as IIb. IC was located in the calf in 11 limbs, the thigh in 8 limbs, and the buttock in 6 limbs.

**Table:** Table 2 Characteristics of symptoms and arteriosclerosis lesions, and results of EVT

	Affected limbs (n = 14)
ABI	0.59 (0.56, 0.8)
Claudication site	
Buttock	6 (43)
Thigh	8 (57)
Calf	11 (79)
Fontaine classification IIa/IIb	11 (79)/3 (21)
TASC classification A/B/C/D	2 (14)/6 (43)/2 (14)/4 (29)
Lesion site	
CIA	6 (43)
EIA	6 (43)
CIA−EIA	2 (14)
Significant stenosis/occlusion	7 (50)/7 (50)
Endovascular therapy	
BMS/stent graft	12 (86)/2 (14)
Operative duration (min)	105 (74, 131)
Bleeding (g)	30 (8, 49)

Data are presented as n (%) or median (interquartile range). ABI: ankle brachial index; BMS: bare metal stent; CIA: common iliac artery; EIA: external iliac artery; EVT: endovascular therapy; TASC: transatlantic inter society consensus

Significant aortoiliac lesions were documented in the common iliac artery (6 limbs), external iliac artery (6 limbs), or both the common and external iliac arteries (2 limbs). No notable lesions were found in the femoropopliteal or infrapopliteal arteries, and below-knee runoff was good in all patients.

Results of EVT are summarized in **Table 2**. All procedures achieved technical success, with no residual stenosis. There were no intraoperative or postoperative complications. During the follow-up period, IC did not worsen, and there was no restenosis of the treated lesions.

### ABI, walking distance, RT, and WIQ score

**Table 3** shows the patients’ ABI, walking distances, RT, and WIQ score at each time point. All parameters changed significantly between the preoperative measurements and the 6-month postoperative time point (Friedman test). In the multiple comparison analysis, ABI increased significantly from preoperative to 1 week postoperative (median 0.59 [IQR 0.56, 0.80]–0.97 [0.91, 1.06]; *P* = 0.0065). There was no difference in ABI between 1 week, 1 month, and 6 months. From preoperative to 1 week postoperative, both PWD (32 [27, 49]–84 [57, 141] m; *P* = 0.01) and MWD (92 [65, 116]–141 [112, 397] m; *P* = 0.0065) increased significantly.

**Table:** Table 3 A summary of the parameters of ABI, treadmill test, and WIQ at preoperative, 1-week, 1-month, and 6-month measurements

	Affected limbs (n = 14)					Multiple pairwise comparison analysis					
	Pre	1 week	1 month	6 months	*P* value^*a*^	Pre–1W *P* value	Pre–1M *P* value	Pre–6M *P* value	1W–1M P value	1W–6M *P* value	1M–6M *P* value
ABI	0.59 (0.56, 0.8)	0.97 (0.91, 1.06)	0.99 (0.90, 1.07)	0.97 (0.87, 1.01)	**<0.001**	**0.0065**	**0.020**	**0.0022**	1	1	1
PWD (m)	32 (27, 49)	84 (57, 141)	111 (61, 345)	100 (57, 359)	**<0.001**	**0.01**	**0.0065**	**0.0065**	1	1	1
MWD (m)	92 (65, 116)	141 (112, 397)	231 (100, 399)	160 (108, 397)	**<0.001**	**0.0065**	**0.0065**	**0.028**	1	1	1
RT (s)	155 (90, 300)	39 (15, 61)	25 (4, 113)	35 (15, 90)	**0.0018**	**0.023**	**0.031**	**0.046**	1	1	1
WIQ score											
Pain	50 (25, 50)	−	100 (100, 100)	100 (75, 100)	**<0.001**	−	**0.015**	**0.024**	−	−	1
Distance	22 (15, 38)	−	100 (72, 100)	100 (45, 100)	**0.004**	−	**0.012**	**0.012**	−	−	1
Walking speed	27 (15, 38)	−	57 (51, 65)	53 (50, 57)	**0.0043**	−	**0.012**	**0.012**	−	−	0.43
Stair climbing	31 (29, 45)	−	77 (57, 97)	77 (47, 100)	**0.031**	−	0.089	0.063	−	−	1

Data are presented as median (interquartile range).

Boldface *P* values represent statistical significance.

^a^A result of the Friedman test.

1M: 1 month; 6M: 6 months; 1W: 1 week; ABI: ankle brachial index; WIQ: WIQ: Walking Impairment Questionnaire; MWD: maximum walking distance; Pre: preoperative; PWD: pain-free walking distance; RT: recovery time

Similarly, RT decreased significantly from preoperative to 1 week (155 [90, 300]–39 [15, 61] s; *P* = 0.023). Walking distance and RT did not change between 1 week, 1 month, and 6 months. WIQ scores improved significantly from preoperative to 1-month measurement in 3 items: walking pain (50 [25, 50]–100 [100, 100]; *P* = 0.015), walking distance (22 [15, 38]–100 [72, 100]; *P* = 0.012), and walking speed (27 [15, 38]–57 [51, 65]; *P* = 0.012). There was no difference in WIQ score between 1 month and 6 months.

In this study, as there was a mix of patients with unilateral and bilateral disease and there were concerns that WIQ scores of individual limbs in patients with bilateral lesions were influenced by the condition of the contralateral limb, we performed a subgroup analysis for the unilateral and bilateral lesion groups, and found no significant differences between the 2 groups (data not shown).

### Spatiotemporal parameters

**Figure 1:**
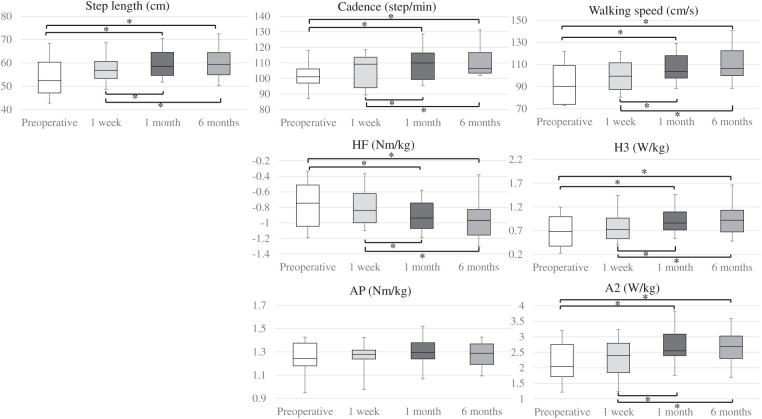
Fig. 1 Results of multiple pairwise comparison analysis of step length, cadence, walking speed, HF, H3, AP, and A2 using the Wilcoxon signed-rank test and Bonferroni *P* value adjustment method. *P < 0.05. A2: ankle plantar flexor muscle; AP: ankle plantar flexor moment; H3: hip flexor muscle; HF: hip flexor moment

**Table 4** reports the spatiotemporal and kinetic parameters at each time point. All spatiotemporal parameters were significantly different at 6 months compared with the preoperative measurement (Friedman test). The results of the multiple comparison analysis are shown in **Fig. 1** (details in **Supplemental Table 1**).

**Table:** Table 4 A summary of biomechanical gait parameters in patients at preoperative, 1-week, 1-month, and 6-month time points, and in control

	Affected limbs (n = 14)					Controls (n = 10)	Patients vs. controls	
	Preoperative	1 week	1 month	6 months	*P* value^a^		Pre vs. Co *P* value	6M vs. Co *P* value
Step length (cm)	52.42 (47.11, 60.33)	56.78 (53.34, 60.64)	58.54 (54.63, 64.54)	59.41 (54.92, 64.43)	**<0.001**	65.24 (64.38, 71.87)	**<0.001**	**0.007**
Cadence (step/min)	101.09 (97.05, 106.12)	109.22 (94.01, 113.66)	109.96 (99.36, 116.42)	106.49 (103.39, 116.67)	**<0.001**	115.8 (110.2, 122.17)	**0.005**	0.24
Walking speed (cm/s)	90.17 (73.98, 108.9)	99.42 (87.38, 111.57)	103.49 (97.66, 117.94)	106.41 (99.85, 122.42)	**<0.001**	129.37 (126, 132.79)	**<0.001**	**0.008**
HE^b^	0.39 (0.3, 0.43)	0.41 (0.34, 0.52)	0.42 (0.38, 0.46)	0.44 (0.4, 0.49)	**0.015**	0.46 (0.41, 0.64)	**0.022**	0.52
HF^b^	−0.75 (−1.04, −0.51)	−0.84 (−1, −0.62)	−0.94 (−1.07, −0.74)	−0.97 (−1.16, −0.83)	**<0.001**	−0.99 (−1.04, −0.9)	0.069	0.77
KE^b^	0.35 (0.25, 0.48)	0.27 (0.25, 0.4)	0.42 (0.33, 0.59)	0.4 (0.28, 0.57)	0.086	0.63 (0.48, 0.81)	**0.03**	0.069
KF^b^	−0.28 (−0.31, −0.16)	−0.25 (−0.28, −0.19)	−0.25 (−0.32, −0.16)	−0.22 (−0.27, −0.2)	0.65	−0.23 (−0.25, −0.18)	0.77	0.82
AD^b^	−0.026 (−0.039, −0.019)	−0.031 (−0.042, −0.026)	−0.033 (−0.037, −0.027)	−0.032 (−0.051, −0.025)	0.41	−0.03 (−0.04, −0.03)	0.11	0.68
AP^b^	1.24 (1.18, 1.37)	1.28 (1.24, 1.31)	1.29 (1.24, 1.38)	1.29 (1.19, 1.37)	**0.04**	1.31 (1.2, 1.39)	0.52	0.86
H1^c^	0.3 (0.22, 0.4)	0.4 (0.25, 0.58)	0.36 (0.21, 0.46)	0.41 (0.27, 0.51)	0.11	0.44 (0.17, 0.49)	0.24	0.82
H2^c^	−0.45 (−0.54, −0.42)	−0.63 (−0.77, −0.41)	−0.82 (−0.93, −0.65)	−0.81 (−1.14, −0.61)	**0.0013**	−1.13 (−1.26, −0.84)	**0.002**	0.069
H3^c^	0.68 (0.38, 1)	0.73 (0.53, 0.96)	0.86 (0.72, 1.1)	0.92 (0.67, 1.13)	**<0.001**	1.01 (0.96, 1.13)	**0.016**	0.38
K1^c^	−0.22 (−0.58, −0.093)	−0.2 (−0.38, −0.081)	−0.4 (-0.7, −0.14)	−0.41 (−0.72, −0.13)	**0.033**	−0.85 (−1.49, −0.38)	**0.01**	**0.022**
K2^c^	0.2 (0.15, 0.4)	0.17 (0.097, 0.32)	0.31 (0.24, 0.48)	0.25 (0.14, 0.43)	**0.047**	0.52 (0.27, 0.99)	**0.035**	0.053
K3^c^	−0.92 (−1.4, −0.46)	−0.89 (−1.26, −0.64)	−1.12 (−1.59, −0.89)	−1.12 (−1.65, −0.88)	**<0.001**	−1.37 (−1.89, −1.24)	**0.026**	0.16
A1^c^	−0.45 (−0.54, −0.42)	−0.53 (−0.55, −0.42)	−0.49 (−0.6, −0.46)	−0.53 (−0.57, −0.46)	0.16	−0.6 (−0.65, −0.4)	0.32	0.48
A2^c^	2.04 (1.71, 2.75)	2.4 (1.85, 2.79)	2.55 (2.39, 3.08)	2.68 (2.3, 3.02)	**<0.001**	2.86 (2.49, 3.06)	**0.04**	0.52

Data are presented as median (interquartile range). Boldface *P* values represent statistical significance.

^a^A result of the Friedman test.

^b^Parameter of joint moment (Nm/kg).

^c^Parameter of joint power (W/kg).

AD: ankle dorsiflexor; AP: ankle plantar flexor; Co: controls; HE: hip extensor; HF: hip flexor; KE: knee extensor; KF: knee flexor

There was no significant difference in step length, cadence, or walking speed at 1 week postoperatively compared with preoperative values. From preoperative to 1 month, there was a significant increase in step length (52.42 [47.11, 60.33]–58.54 [54.63, 64.54] cm; *P* = 0.0037), cadence (101.09 [97.05, 106.12]–109.96 [99.36, 116.42] step/min; *P* = 0.024), and walking speed (90.17 [73.98, 108.9]–103.49 [97.66, 117.94] cm/s; *P* = 0.0022). These parameters did not change between 1 month and 6 months.

### Kinetic parameters

HE, HF, AP, H2, H3, K1, K2, K3, and A2 all changed significantly from preoperative to 6 months postoperative (Friedman test; **Table 4**). The results of the multiple comparison analysis are shown in **Fig. 1** and **Supplemental Table 1**. There were no differences in any kinetic parameters between preoperative and 1 week postoperative.

Considering joint moment, HE (0.39 [0.3, 0.43]–0.42 [0.38, 0.46] Nm/kg; *P* = 0.018) and HF (−0.75 [−1.04, −0.51] to −0.94 [−1.07, −0.74] Nm/kg; *P* = 0.04) significantly increased from preoperative to 1 month, and there were no significant differences between 1 month and 6 months in these parameters. AP, despite showing overall significance in the Friedman test, did not show significant differences in the multiple comparison analysis.

Considering joint power, there was a significant increase from preoperative to 1 month postoperative, especially in H2 (−0.45 [−0.54, −0.42] to −0.82 [−0.93, −0.65] W/kg; *P* = 0.04), H3 (0.68 [0.38, 1]–0.86 [0.72, 1.1] W/kg; *P* = 0.018), and A2 (2.04 [1.71, 2.75]–2.55 [2.39, 3.08] W/kg; *P* = 0.0015). There were no significant differences in joint power between 1 month and 6 months.

### Comparison with healthy controls

We compared biomechanical parameters between patients before surgery and 6 months postoperatively with healthy controls (**Table 4** and **Supplemental Figs. 3–5**). Compared to controls, preoperative patients had significant decline in all spatiotemporal parameters: step length (52.42 [47.11, 60.33] vs. control 65.24 [64.38, 71.87] cm; *P* < 0.001), cadence (101.09 [97.05, 106.12] vs. 115.8 [110.2, 122.17] step/min; *P* = 0.005), and walking speed (90.17 [73.98, 108.9] vs. 129.37 [126, 132.79] cm/s; *P* < 0.001). Similarly, patients were significantly worse than controls in HE (0.39 [0.3, 0.43] vs. 0.46 [0.41, 0.64] Nm/kg; *P* = 0.022), KE (0.35 [0.25, 0.48] vs. 0.63 [0.48, 0.81] Nm/kg; *P* = 0.03), and most parameters of joint power: H2 (−0.45 [−0.54, −0.42] vs. −1.13 [−1.26, −0.84] W/kg; *P* = 0.002), H3 (0.68 [0.38, 1] vs. 1.01 [0.96, 1.13] W/kg; *P* = 0.016), A2 (2.04 [1.71, 2.75] vs. 2.86 [2.49, 3.06] W/kg; *P* = 0.04), and others (**Table 4**).

At 6 months postoperative, step length (59.41 [54.92, 64.43] cm; *P* = 0.007) and walking speed (106.41 [99.85, 122.42] cm/s; *P* = 0.008) remained significantly lower than controls, but there were no significant differences from controls in most kinetic parameters.

## Discussion

To our knowledge, few studies have evaluated gait biomechanics after revascularization in patients with IC. The present prospective cohort study supports the hypothesis that EVT used to treat aortoiliac artery lesions improves gait biomechanical abnormalities caused by HF muscle dysfunction.

We previously found that hip flexor muscle dysfunction is reflected by a decline in peak HF and H3.^11)^ HF is mainly caused by contraction of the iliopsoas muscle, while H3 is generated in the late stance phase to raise the thigh and contributes to advancement of the body.^13)^ The iliopsoas muscle is supplied by the iliolumbar artery and medial femoral circumflex artery, which are branches of the internal iliac artery and deep femoral artery. An improvement in blood flow to the iliopsoas muscle following EVT may explain the improved HE, H3, and other spatiotemporal parameters described in the present study.

Previous studies of gait in patients with LEAD focused on the reduction in AP and A2.^6,7)^ In the present study, A2 was significantly increased despite there being no significant change in AP in the multiple comparison analysis (unlike HF and H3, which significantly improved a month after EVT). This suggests that the increase in A2 was due to an increase in angular velocity rather than the joint moment, perhaps due to improvements in other biomechanical parameters such as walking speed, HF, and H3.

Interestingly, biomechanical parameters took a month to improve, despite the fact that improvements in ABI, PDW, MWD, and RT were recorded only 1 week after EVT. This discrepancy may be explained by the continuing improvement of blood flow and relief from pain, and may be related to the patients’ muscle condition. Previous studies have shown that muscle dysfunction in patients with LEAD is caused by weakness, atrophy, fatty degeneration, metabolic myopathy, and polyneuropathy of muscle due to ischemia.^19–22)^ Improvement in blood flow is expected to change muscle condition over time. However, as we did not evaluate muscle strength, physiological changes, or metabolic changes, we cannot say with certainty that this explains the late improvement in biomechanical parameters observed in our study.

We further showed no change in any biomechanical parameter from 1 month to 6 months after EVT. This result demonstrates that the improvement achieved at 1 month is maintained for at least 6 months after surgery, but also that no further improvement is achieved. Future studies should investigate the long-term durability of these biomechanical gait improvements after EVT.

We included matched healthy controls in this study to clarify the weakness of treated patients before and 6 months after EVT. Before EVT, we found significant decline in all spatiotemporal parameters, HE, KE, and most joint power parameters. Six months after EVT, step length, and walking speed were still significantly poorer than those of healthy controls, but most kinetic parameters were equivalent to those of healthy controls. The improvement in kinetic parameters suggests that the gait patterns in patients after treatment are similar to those of healthy controls, but the step length and walking speed suggest that additional treatment, such as exercise, is required along with EVT.

Several studies using 3D motion analysis after supervised exercise therapy reported changes in biomechanical parameters (muscle strength and kinetic parameters) after treadmill walking exercise^10)^ or bicycle ergometer exercise,^9)^ but reported no change in (or did not record) step length and walking speed.

However, previous studies in elderly subjects reported that resistance training, such as leg press, knee flexion and extension, hip abduction and adduction, and ankle plantar flexion and dorsiflexion, improved stride length and walking speed.^23,24)^ The effect of resistance training in patients with LEAD is still unclear due to a limited number of published studies and the disparate outcome methodologies used,^25)^ but when used in combination with EVT, it may be expected that the patients achieve a gait closer to normal. In addition, we found that these parameters improved a month after EVT but did not further improve, so it may be necessary to intervene at the latest a month after EVT.

This study had several limitations. First, the number of subjects was only 14 limbs (10 patients), which may not have been sufficient for detailed statistical analysis. Second, our findings were limited to patients with isolated aortoiliac artery lesions. Gait biomechanics associated with femoropopliteal artery lesions, infrapopliteal artery lesions, and complex lesions remain to be investigated. Third, we included patients with both unilateral and bilateral lesions. The presence of a lesion-free limb may affect gait biomechanical parameters, such as masking symptom improvement in patients with unilateral lesions. Furthermore, due to the small sample size, we could not analyze patients with unilateral and bilateral lesions separately. Fourth, we evaluated gait biomechanical parameters using patient-selected walking speed. Gait evaluation performed at a fast walking pace is known to emphasize the patients’ potential ability.^26)^ Fifth, we did not evaluate muscle condition and physical activity. Future use of these parameters may allow more accurate assessment of the effects of EVT.

## Conclusion

In conclusion, the present prospective cohort study indicated that EVT for aortoiliac artery lesions improved spatiotemporal parameters (step length, cadence, and walking speed) and kinetic parameters (especially HF and H3 related to hip flexor muscle) a month after EVT, and that this improvement was maintained for 6 months.

The gait pattern of patients 6 months after EVT was close to that of healthy controls, but step length and walking speed remained low. Further studies are needed to investigate the long-term durability and additional treatment strategies, such as exercise therapy, to further improve gait in patients with IC.

## Declarations

### Disclosure statement

The authors have no conflicts of interest to report.

### Author contributions

Study conception: TKak, MK

Data collection: NO, TKak

Analysis: NO, TKak

Investigation: NO, TKak, MK

Manuscript preparation: NO, TKak

Funding acquisition: TKak, MK

Critical review and revision: all authors

Final approval of the article: all authors

Accountability for all aspects of the work: all authors.

## Supplementary Materials

Supplementary Fig. 1 Reflective markers were attached to 17 anatomical landmarks on the participants as suggested by the Clinical Gait Analysis Forum of Japan: the right and left acromion, anterior and posterior superior iliac spine, greater trochanter, femoral lateral epicondyle, lateral malleolus, fifth metatarsal head, calcaneus, and the right angulus inferior scapulae.

Supplementary Fig. 2 Typical graph of joint moment and joint power at the hip, knee, and ankle in the sagittal plane during the stance phase. (**A**) Joint moment is shown for HE and HF, KE and KF, and AD and AP during the stance phase. (**B**) Joint power is shown. Hip power generation during loading response is produced by concentric contraction of the hip extensor muscle (H1); hip power absorption during mid stance is due to eccentric contraction of the hip flexor muscle (H2); hip power generation during preswing is produced by concentric contraction of the hip flexor muscle (H3); knee power absorption during loading response is due to eccentric contraction of the knee extensor muscle (K1); knee power generation during mid stance is produced by concentric contraction of the knee extensor muscle (K2); knee power absorption during preswing is due to eccentric contraction of the knee extensor muscle (K3); ankle power absorption during mid stance and terminal stance is due to eccentric contraction of the ankle plantar flexor muscle (A1); and ankle power generation during preswing is produced by concentric contraction of the ankle plantar flexor muscle (A2). Words written in the graph are mean peak values: 0% indicates the start of the stance phase (initial contact) and 100% indicates the end of the stance phase (preswing). AD: ankle dorsiflexor; AP: ankle plantar flexor; HE: hip extensor; HF: hip flexor; KE: knee extensor; KF: knee flexor. This figure was reproduced from J Vasc Surg^11)^. Copyright 2017.

Supplementary Fig. 3 Comparison of spatiotemporal parameters between patients before surgery and 6 months postoperative with healthy controls. *P < 0.05.

Supplementary Fig. 4 Comparison of joint moment between patients before surgery and 6 months postoperative with healthy controls. *P < 0.05. AD: ankle dorsiflexor; AP: ankle plantar flexor; HE: hip extensor; HF: hip flexor; KE: knee extensor; KF: knee flexor

Supplementary Fig. 5 Comparison of joint power between patients before surgery and 6 months postoperative with healthy controls. *P < 0.05.

Supplementary Table 1 Results of multiple pairwise comparison analysis of biomechanical parameters that showed a significant difference in the Friedman test.
