# Supplementary material for: Improvement of Gait Biomechanics after Endovascular Therapy for Patients with Intermittent Claudication Associated with Aortoiliac Occlusive Disease
Source: Ann Vasc Dis. 2025 Jun 10;18(1):25-00006. doi: 10.3400/avd.oa.25-00006 (PMC12158554; doi:10.3400/avd.oa.25-00006)

Supplemental Fig. 1

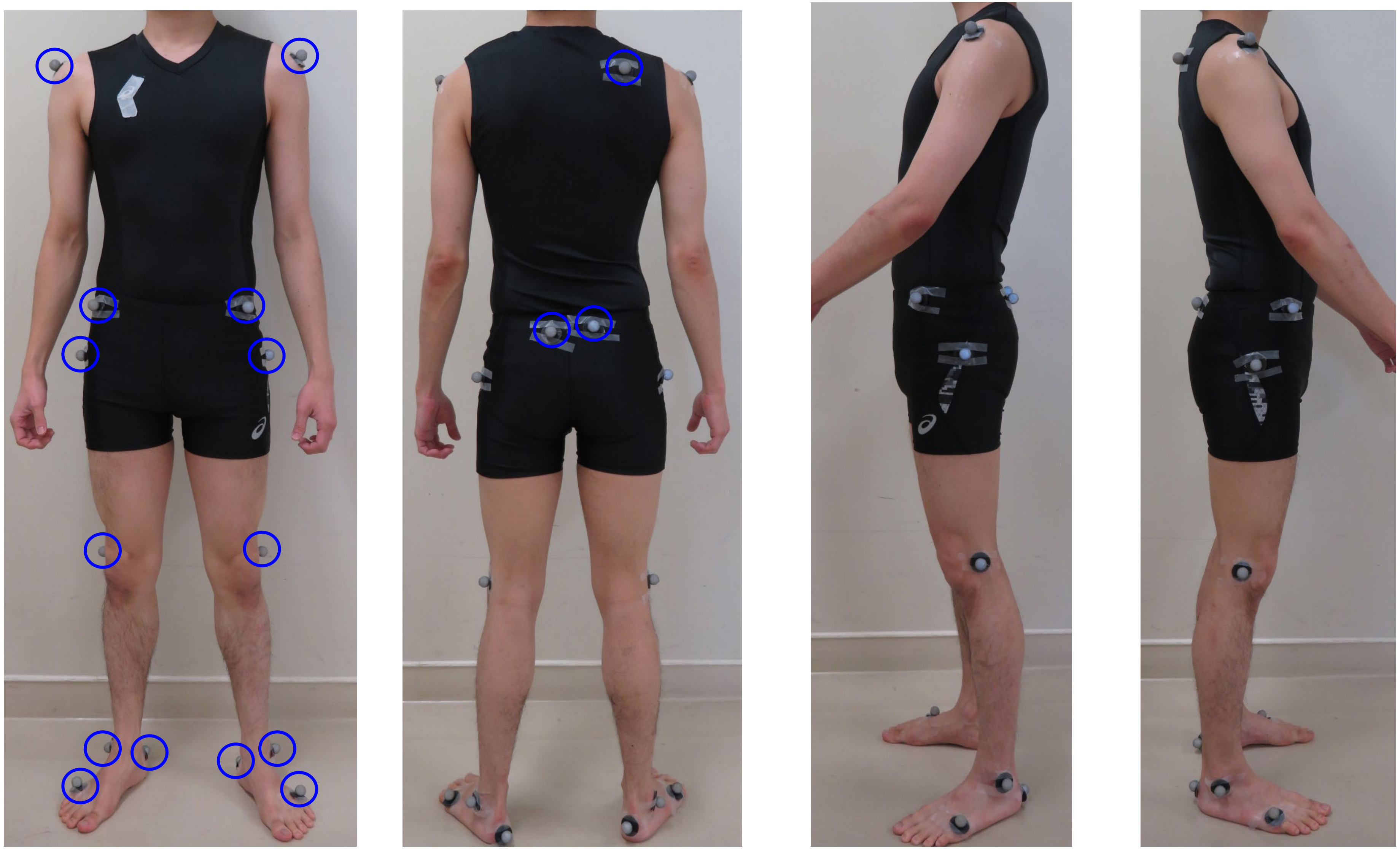

Supplemental Fig. 2

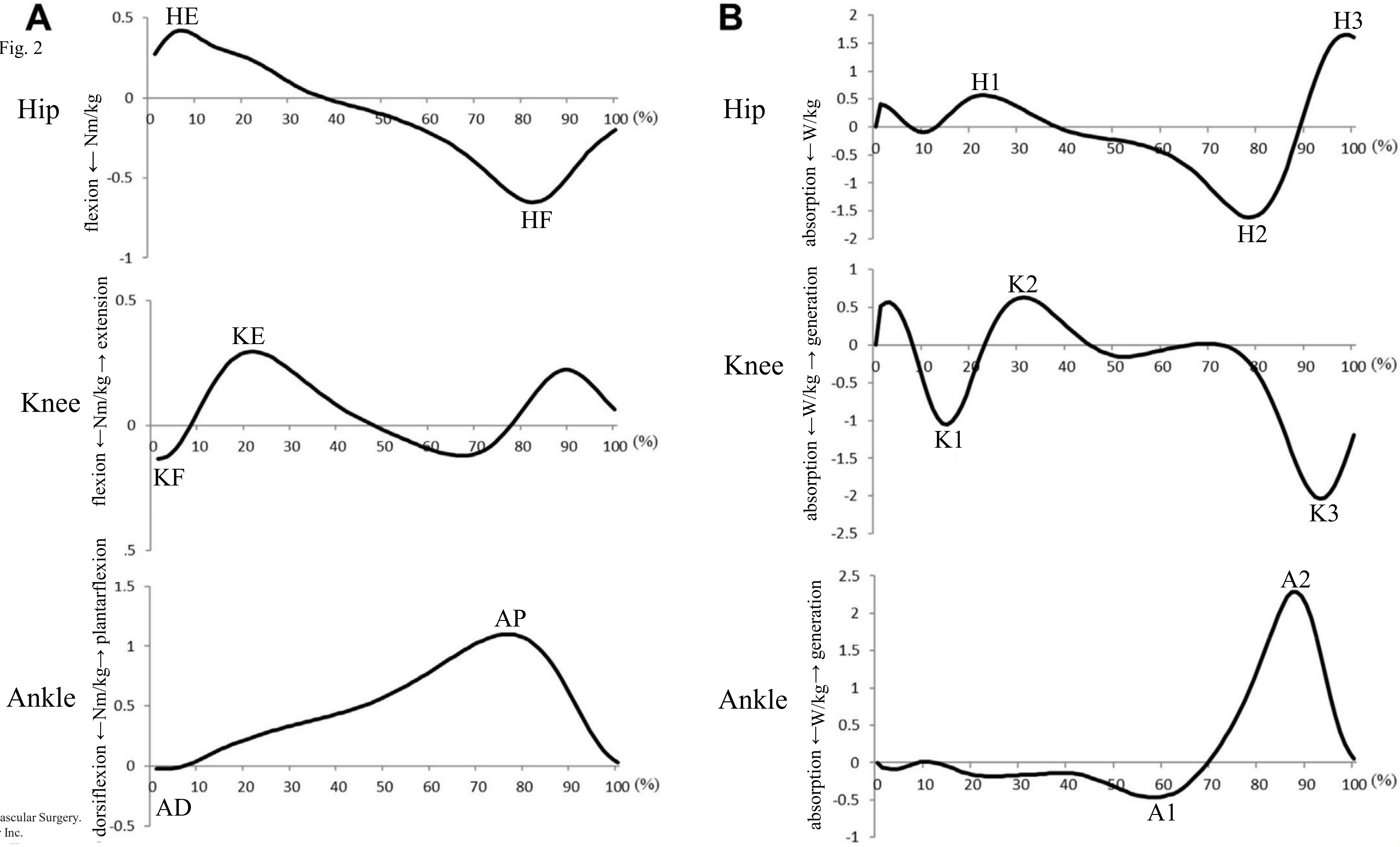

© 2017 Society for Vascular Surgery.
Published by Elsevier Inc.

Supplemental Fig. 3

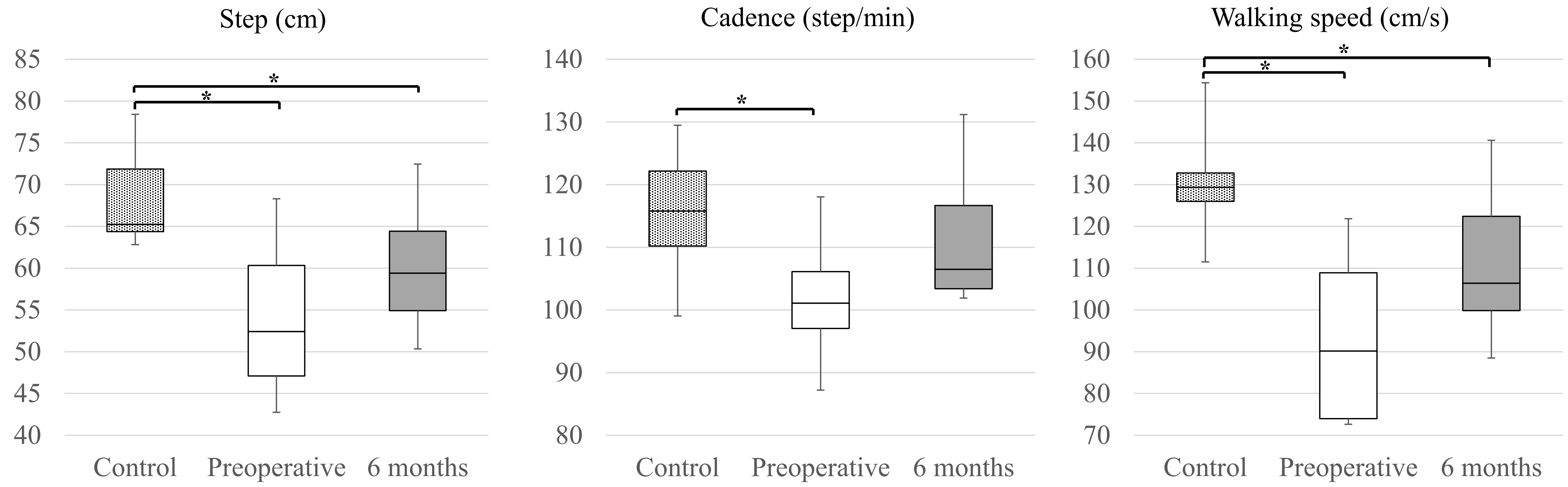

Supplemental Fig. 4

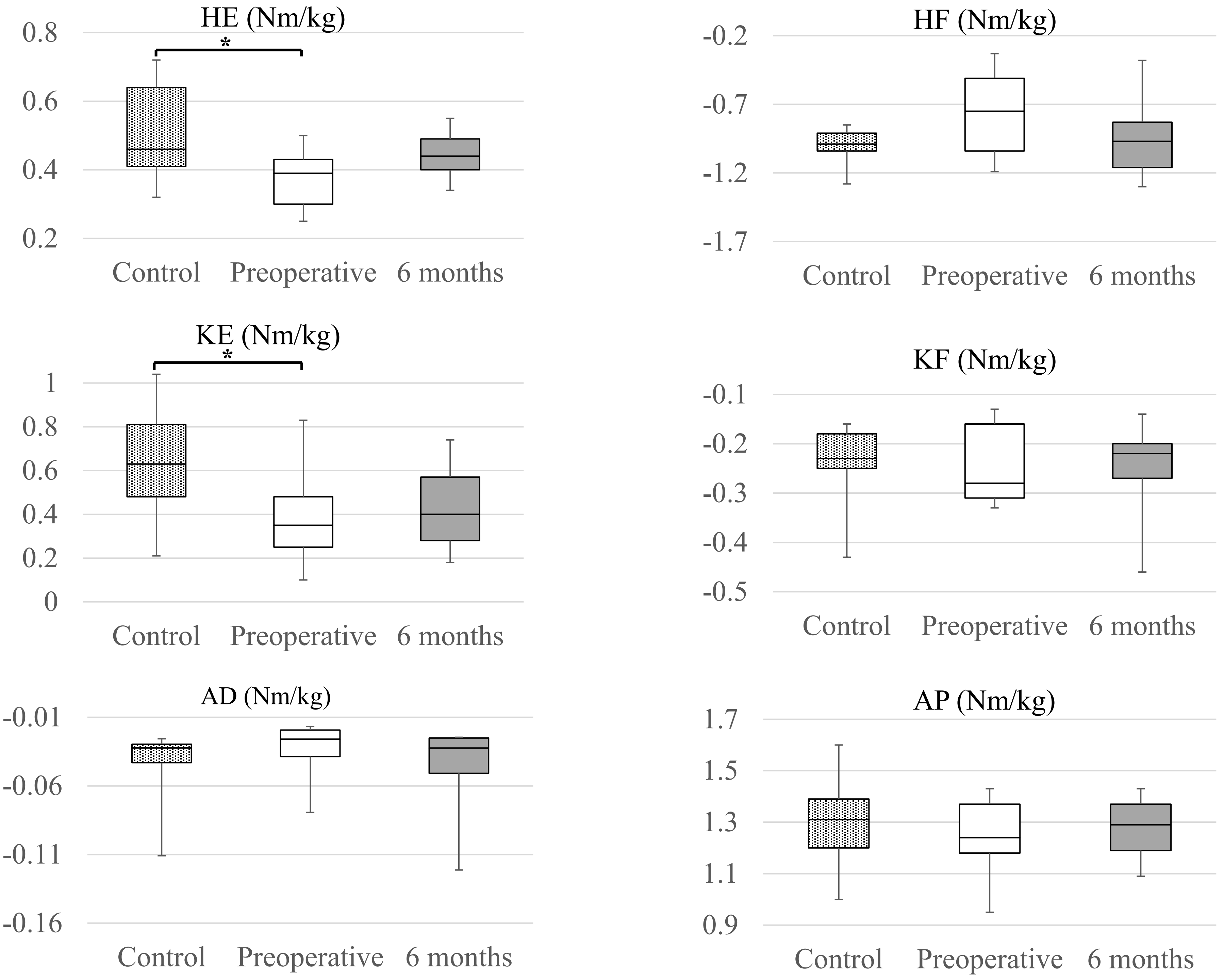

Supplemental Fig. 5

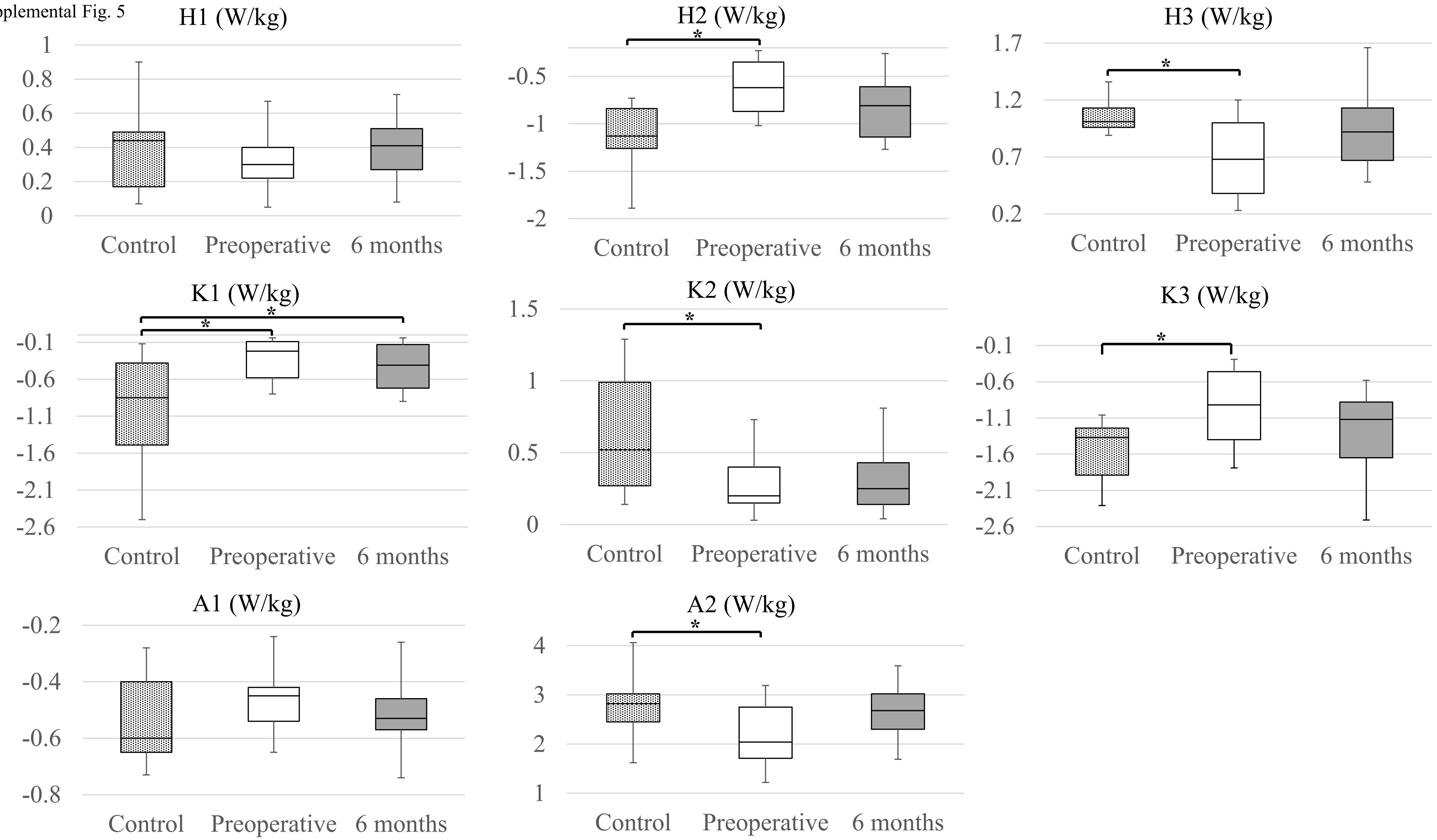

Supplement: Supplementary Fig. 1 — Reflective markers were attached to 17 anatomical landmarks on the participants as suggested by the Clinical Gait Analysis Forum of Japan: the right and left acromion, anterior and posterior superior iliac spine, greater trochanter, femoral lateral epicondyle, lateral malleolus, fifth metatarsal head, calcaneus, and the right angulus inferior scapulae. [file avd-18-1-25-00006-s01.pdf]
