# Supplementary material for: Improvement of Gait Biomechanics after Endovascular Therapy for Patients with Intermittent Claudication Associated with Aortoiliac Occlusive Disease
Source: Ann Vasc Dis. 2025 Jun 10;18(1):25-00006. doi: 10.3400/avd.oa.25-00006 (PMC12158554; doi:10.3400/avd.oa.25-00006)
Supplement: Supplementary Table 1 — Results of multiple pairwise comparison analysis of biomechanical parameters that showed a significant difference in the Friedman test. [file avd-18-1-25-00006-s02.pdf]

Supplemental Table 1 Results of multiple pairwise comparisons analysis of biomechanical parameters which showed a significant difference in the Friedman test

	Patients (n = 14)					
	Pre – 1W	Pre – 1M	Pre – 6M	1W – 1M	1W – 6M	1M – 6M
	P value	*P* value	*P* value	*P* value	*P* value	*P* value
Step length, cm	.1	**.0037**	**.0022**	**.014**	**.01**	1
Cadence, step/min	1	**.024**	**.0051**	**.018**	**.014**	.25
Walking speed, cm/s	.62	**.0022**	**.0022**	**.0037**	**.0015**	.35
HE[a]	.41	**.018**	**.018**	1	1	1
HF[a]	1	**.04**	**.0073**	**.0015**	**.01**	1
AP[a]	1	.54	1	.35	1	1
H2[b]	1	**.04**	**.0073**	**.014**	**.01**	1
H3[b]	1	**.018**	**.0037**	**.04**	**.0051**	1
K1[b]	1	.71	.41	**.04**	.08	1
K2[b]	1	.21	1	.18	.25	.47
K3[b]	1	**.024**	.08	**.024**	**.0073**	1
A2[b]	1	**.0015**	**.0051**	**.01**	**.0073**	1

Boldface *P* values represent statistical significance.

a Parameter of joint moment (Nm/kg).

b Parameter of joint power (W/kg)

Pre: preoperative; 1W: 1 week; 1M: 1 month; 6M: 6 months; Co: control; HE: hip extensor; HF: hip flexor; KE: knee extensor; AP: ankle plantar flexor
